# Glutaminolysis: A Driver of Vascular and Cardiac Remodeling in Pulmonary Arterial Hypertension

**DOI:** 10.3389/fcvm.2021.667446

**Published:** 2021-04-28

**Authors:** Richard Mprah, Gabriel Komla Adzika, Yusif I. Gyasi, Marie Louise Ndzie Noah, Joseph Adu-Amankwaah, Adebayo O. Adekunle, Maxwell Duah, Prosperl Ivette Wowui, Qiao Weili

**Affiliations:** ^1^Department of Physiology, Xuzhou Medical University, Xuzhou, China; ^2^Department of Chemistry & Biochemistry, Central Michigan University, Mount Pleasant, TX, United States; ^3^Haematology Department, Affiliated Hospital of Xuzhou Medical University, Xuzhou, China; ^4^School of Nursing, Xuzhou Medical University, Xuzhou, China

**Keywords:** pulmonary arterial hypertension, metabolic dysregulation, glutaminolysis, vascular and cardiac remodeling, endothelial dysfunction

## Abstract

Pulmonary arterial hypertension (PAH) is a decimating ailment described by chronic precapillary pulmonary hypertension, an elevated mean pulmonary arterial pressure with a normal pulmonary capillary wedge pressure, and a raised pulmonary vascular resistance resulting in increased right ventricular afterload culminating in heart failure and death. Current PAH treatments regulate the vasodilatory/vasoconstrictory balance of pulmonary vessels. However, these treatment options are unable to stop the progression of, or reverse, an already established disease. Recent studies have advanced a metabolic dysregulation, featuring increased glutamine metabolism, as a mechanism driving PAH progression. Metabolic dysregulation in PAH leads to increased glutaminolysis to produce substrate to meet the high-energy requirement by hyperproliferative and apoptosis-resistant pulmonary vascular cells. This article explores the role of glutamate metabolism in PAH and how it could be targeted as an anti-remodeling therapeutic strategy.

## Introduction

Pulmonary hypertension (PH) entails a group of pulmonary vascular diseases exhibiting a common feature: a high mean pulmonary arterial pressure (≥25 mm Hg at rest). PH has been, on the etiological basis, categorized into five main groups. Pulmonary arterial hypertension (PAH), classified under group 1 PH, is rare but the most decimating type of PH, which affects 15–50 people per million (1). The defining characteristics of PAH constitute abnormal hemodynamics as reviewed by other researchers (1, 2), resulting in loss of exercise capacity, breathlessness, right ventricular failure (RVF), and ultimately death. Pathologically, PAH is depicted by inflammation, excessive remodeling of pulmonary blood vessels, obstruction of pulmonary arteries, and increment in pulmonary vascular resistance (PVR), resulting in a pronounced rise in afterload of the right ventricle (RV) (3, 4). Consequently, the RV cannot cope with the increase in load, and heart failure (HF) develops (1). Currently, PAH is treated with soluble guanylate cyclase (SGC) stimulators, endothelin receptor antagonists, phosphodiesterase-5 (PDE5) inhibitors, prostacyclin (PGI_2_) analogs, and PGI_2_ receptor agonists (5–8). Even though these drugs have helped improve PAH patients' quality of life and decrease PVR via acting on the balance between vasoconstriction and vasodilation, they have not been successful at halting the progression, reversing, or curing the disease (2, 4, 9, 10). Progressive pulmonary vascular obstruction leading to increased PVR, which is the mechanism responsible for the HF observed in PAH, may be driven by endothelial cell (EC) dysfunction, cancer-like proliferation, apoptosis-resistant vascular cells, and dysregulation of vascular cell metabolism (3, 11). Recent researches have suggested metabolic reprogramming as a mechanism underlying the hyperproliferative and apoptosis-resistant pulmonary vascular cell (PVC) phenotypes associated with vascular remodeling in PAH (12). This review, therefore, elucidates metabolic reprogramming in PAH, with emphasis on how glutamate (Glu) provides a substrate that fuels energy production to drive vascular and cardiac remodeling in PAH, thereby exacerbating the progression of the disease. It also sheds light on the possibility of targeting Glu and its receptors as a therapeutic effector in PAH treatment.

## Pathobiology Of PAH

Remodeling of pulmonary vessels, which often occurs in the pulmonary arterioles, is a distinguishing characteristic of many forms of PH. Pulmonary vascular lesions, the precursor of vascular remodeling, occurring in both human PAH patients and animal models of PH occur sequentially. They entail, with some variations, atypical muscularization of medial and distal precapillary arteries, loss of precapillary arteries, inspissation of the pulmonary arteriole wall with eccentric or concentric laminar lesions, neointimal formation, fibrinoid necrosis, and the formation of plexiform lesions (13, 14). The remodeling process involves changes in the intima, media, and adventitia walls of the blood vessel. These are due to inflammation, hyperplasia, cellular hypertrophy, metabolic dysregulation, abnormality in cell differentiation and apoptosis, and excessive accumulation and migration of extracellular matrix (ECM) components. Even though the causal pathogeneses of PAH remain unclear, many research findings have reported numerous contributing and disease-predisposing factors, such as gene mutations, pulmonary endothelial dysfunction, inflammation, and abnormal cell proliferation in the walls of blood vessels (5, 13–15). Recently, the novel cancer-like hypothesis (metabolic theory) of PAH has been postulated and has its origins in fascinating *in vitro* and *in situ* observations (16, 17). These observations include monoclonal EC expansion observed in idiopathic PAH (iPAH) when compared with ECs reported in lungs of congenital heart disease (CHD) patients (13); evidence of short DNA microsatellite sequence instability within plexiform lesions in iPAH (18); somatic chromosomal abnormalities present in the lungs of PAH patients and cultured cells (19); smooth muscle cells (SMCs) and pulmonary ECs obtained from patients with PAH when taken from their *in vivo* environment maintained their aberrant increased-proliferative and apoptosis-resistant phenotype for more extended periods than control cells (19–21); and human PVCs from PAH patients exhibited a metabolic dysregulation *in situ* and *in vitro* (22–25). Despite this cancer-like concept, there are decisive disparities between the pathogenesis of PAH and carcinogenesis. It is, however, clear that the cancer-like mechanism alone cannot fully elucidate PAH. However, it has uncovered a new field of research regarding the potential use of antiproliferative or cancer drugs in PAH treatment.

## Metabolic Basis of PAH

The metabolic basis of PAH stems from numerous cellular and molecular mechanisms reported in both PH and cancer that depend on the mitochondria as the focal point of metabolism. As many advances are being made in the area of dysregulations in metabolism in PAH, PH/PAH's metabolic theory has, as well, widened and evolved farther away from the tenets of the Warburg effect. New additions to the metabolic theory include dysregulation of many pathways, which is also complicated by evidence of different genotypes that alter these responses. Also, the anatomical focal point of metabolic dysregulation has widened. Earlier hypotheses presumed that metabolic dysregulation would, in a logical manner, occur in the pulmonary vasculature. However, increasing proof indicates a deviation of metabolism inside the RV and may be in muscle, indicating paracrine and system outcomes in PAH progression (26, 27). The essence of metabolic dysfunction beyond the vasculature of the lungs has implications in redefining the understanding of metabolism in PAH. The metabolic principles would be necessary for formulating a more cohesive and comprehensive metabolic theory of PAH. Beyond hyperproliferative and antiapoptotic PVC variants, the increasing scope of metabolic dysregulation occasioned the unearthing of other phenotypes altered by metabolic reprogramming in PAH, such as vessel stiffening, fibrosis, and angiogenesis. Besides cardiomyocytes, ECs, and SMCs, where substantial amounts of the early observations of metabolic dysfunction in PAH cantered, the role of metabolic dysregulation is extending into other types of cells in the vasculature (28). The progressive liaison of metabolic dysregulation and its reprogramming remains a vital question still unanswered. Whether metabolic dysregulation occurs early or late in the progression of PAH is paramount in comprehending the overwhelming scope of metabolic dysfunction in the disease. There are several organized tiers of mitochondrial dysregulation that results in the various cellular phenotypes of PH. The proliferation of PVCs, ECs, fibroblasts, and SMCs is the defining characteristic of the pathology of PH (29). The noticed metabolic repositioning toward glycolysis (Warburg effect) in PAH has been related to varied molecular events that bestow the ability to adapt to acute bouts of cellular stress and resist apoptosis. These molecular events include the activation of master regulatory factors, e.g., hypoxia-inducible factor (HIF) and nuclear factor of activated T cells, bioactive metal homeostasis dysregulation, compensatory anaplerosis, decreased mitochondrial reactive oxygen species, internalization and inhibition of oxygen-sensitive voltage-gated potassium (K^+^) channels, hyperpolarization of mitochondrial membrane, dysregulation of calcium (Ca^2+^) dynamics, endoplasmic reticulum stress, and histone acetylation suppression (29–31). Notwithstanding these short-lived variations, the cell must continue to generate energy and cellular mass at levels enough to meet metabolic requirements, which ideally cannot be solely sustained via enhanced glycolysis and thus require several levels of molecular reprogramming. In the next section, we review how increased Glu uptake provides substrate to meet the high metabolic demands in PAH as a furtherance of the metabolic basis of PAH.

### Implication of Glutamine in PAH

PAH is principally propelled by the increased proliferation and migration of vascular cells, resulting in lesion formation that obstructs the pulmonary blood vessels (32, 33). This abnormal vascular remodeling response accompanied by fibrosis and vasoconstriction results in increased pressure in pulmonary arteries and, ultimately, RVF and premature death. PAH is initiated by injuries due to exogenous substances such as drugs, infections, toxins, hypoxia, CHD, and several mutations, such as bone morphogenetic protein receptor 2 (BMPR2) gene mutation. Whereas, mitochondrial dysfunction and metabolic reprogramming are known to impart the cellular attributes of PAH, the relevance of glutamine metabolism is now emerging. ECM stiffness, an early pathologic event in PAH, has been noted to stimulate SMCs and pulmonary EC proliferation via the induction of glutaminase (GLS1) by Yes-associated protein 1 (YAP) with PDZ-binding motif (TAZ) (34). GLS1 expression has been observed to increase in pulmonary arterioles of the monocrotaline (MCT)–induced PAH rat model, and glutamine measured in isolated pulmonary ECs decreases, suggestive of both increased glutaminolysis and anaplerotic flux through the Krebs cycle (34). The increased glutaminolysis in PAH also enhances fibrosis by inducing the stability and translation of collagen via α-ketoglutarate-mediated mammalian target of rapamycin (mTOR) activation and hydroxylation of proline (35), igniting an aggressive hyperproliferation and arterial stiffening. Pharmacological inhibition of GLS1 activity is reported to disturb this cycle and diminishes arterial remodeling in MCT-induced PAH in rats (36). A similar elevation in GLS1 expression was reported in rhesus macaques monkey with simian-immunodeficiency virus–associated PAH and in the lungs of human immunodeficiency virus–mediated PAH patients (37). The reconfiguration of Glu metabolism contributes to the maladaptive cardiac remodeling response in PAH, as elevation in glutaminolysis has been observed in the RV of both PAH patients and MCT-treated rats (36). Recent studies revealed that PAH patients with abnormal BMPR2 functions showed an enormous diminution in glutamine across the transpulmonary gradient compared to control groups (38), alluding that mutations in this receptor may impinge glutamine metabolism. BMPR2 mutant ECs were also reported to depict a hyperproliferative phenotype but are utterly not tolerant of Glu-limiting conditions (39). Yelamanchi et al. reported that this glutamine-addiction is driven downstream from BMPR2 via oxidant damage of the mitochondria, resulting in the development of isoketals that stabilize HIF-1 and inactivate sirtuin-3 (39). Furthermore, scavenging isoketals normalizes glutamine metabolism and prevents PAH in mice with BMPR2 mutation (39). Thus, therapeutic targeting of glutamine metabolism represents a promising approach in treating various forms of PAH.

### Metabolic Dysregulation and Increased Glutaminolysis in PAH

Metabolic dysregulation has been postulated to be liable for the increased proliferation and apoptosis-resistant phenotypes of PVCs concomitant with the vascular remodeling in PAH (12, 25, 29). This metabolic dysregulation yields a cancer-like glycolytic shift from the normal oxidative phosphorylation (OXPHOS) toward aerobic glycolysis (Warburg effect), elevations in fatty acid oxidation, and glutaminolysis (12, 24, 34, 40). However, a metabolic alteration from OXPHOS to glycolysis alone might not serve as a means of energy (ATP) production for the excessive proliferation of PVCs. Whereas energy generation is less useful for glycolysis per glucose molecule, some earlier studies posited that enough glucose is available in the pulmonary vessels sufficient for ATP production to drive cellular proliferation (31). Nonetheless, a decrease of carbon intermediates, independent of ATP production, conjointly occurs if glucose is jolted away from the tricarboxylic acid (TCA) cycle, hence negatively impacting the carbon substrates employed in the production of nucleotides and proteins (41). Anaplerosis (the process by which carbon intermediates of the TCA cycle are replenished) is crucial in preserving cell mass (nucleotides and proteins), mainly if glycolysis is predominant. One of the pathways for refilling carbon intermediates is through glutaminolysis. Glutaminolysis is an anaplerotic reaction whereby the carbon intermediates of the TCA cycle are refilled, especially when swiftly dividing cells need considerable biomass. Glutaminolysis provides TCA intermediates, which impart fatty and amino acids, and *de novo* biosynthesis of purine and pyrimidine (42). The elevation in glutaminolysis involves GLS1 upregulation and glutamine uptake by the PAH vasculature, leading to increased production of Glu by the PVCs, driving experimental PH (43).

**Figure 1:**
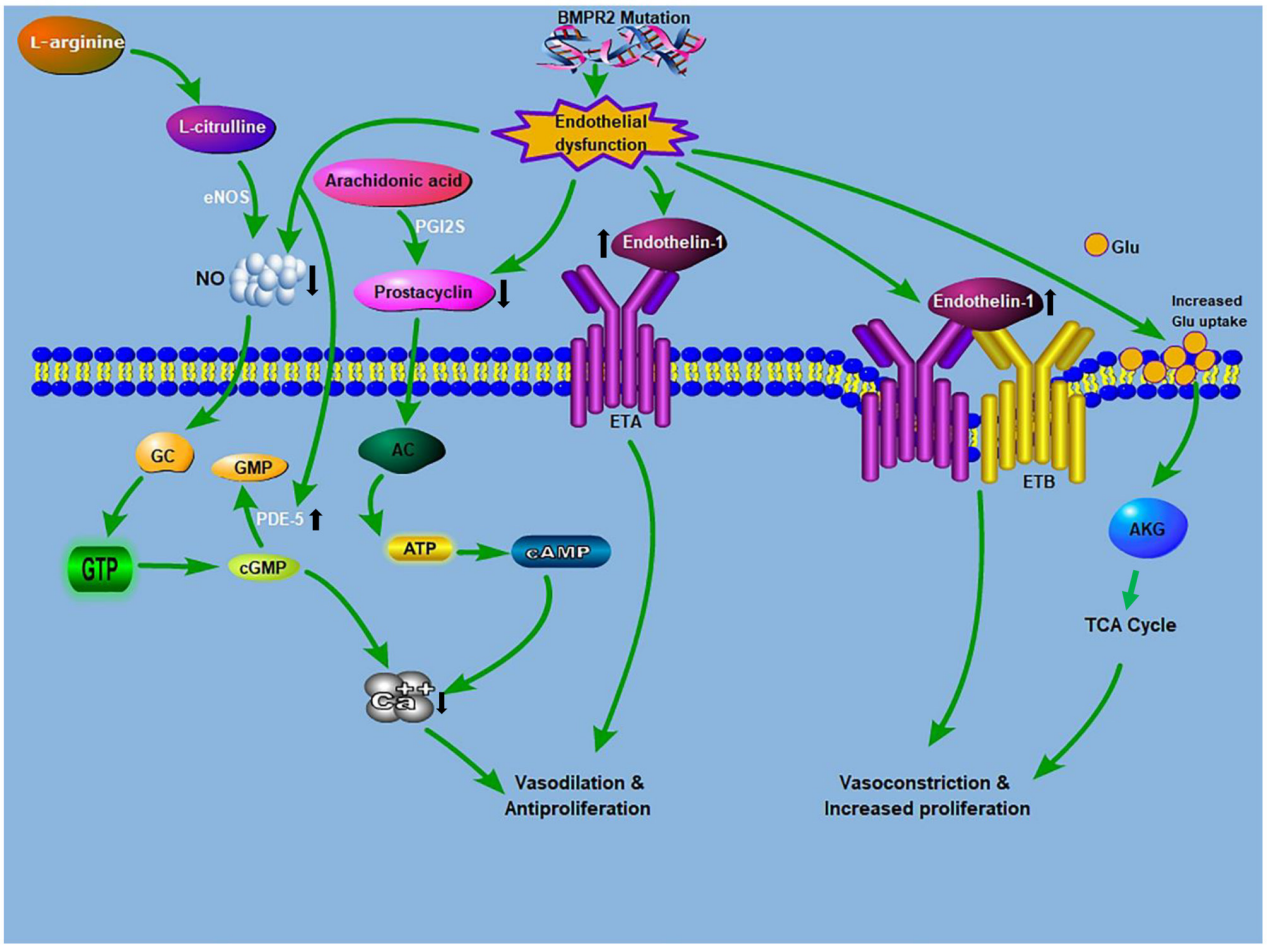
Crosstalk between glutaminolysis and the known PAH pathogenesis: The known PAH pathogenesis: NO, produced from l-arginine, stimulates cGMP production. cGMP causes relaxation and inhibition of vascular SMCs proliferation. PDE5 inhibitors augment the vasodilatory mechanism through the prevention of cGMP deterioration. Prostacyclin from ECs promotes relaxation and inhibition of cell proliferation via a cAMP-dependent mechanism. Endothelin is an effective vasoconstrictor that induces proliferation via ET_A_ receptors on SMCs, while stimulating NO and prostacyclin discharge via endothelial ET_B_ receptors. Glutamate provides carbon intermediates (αKG) for the TCA cycle, thereby promoting cellular ATP production, which culminates in increase proliferation and development of antiapoptotic phenotypes. BMPR2 mutation drives endothelial dysfunction, which causes increased endtholin-1 levels and increases glutamate uptake in endothelial cells. AC, adenylyl cyclase; ATP, adenosine triphosphate; AKG, α-ketoglutarate; BMPR2, bone morphogenetic protein receptor 2; ETA, endothelin receptor A; ETB, endothelin receptor B; cAMP, cyclic adenosine monophosphate; cGMP, cyclic guanosine monophosphate; eNOS, endothelial nitric oxide synthase; GTP, guanosine triphosphate; GC, guanylyl cyclase; Glu, glutamine; NO, nitric oxide; PDE-5, phosphodiesterase 5; PGI2S, prostacyclin synthase; TCA, tricarboxylic acid.

In similitude to cancer cells, PVCs in PAH by anaplerosis utilize Glu (in glutaminolysis) to produce α-ketoglutarate for the TCA cycle (34, 38, 44, 45) (Figure 1). Dysregulation of glutaminolysis in the heart was observed in right ventricular hypertrophy in animal models of PAH (36). A recent study showed that YAP and transcriptional coactivator with a TAZ are essential for glutaminase upregulation and a resultant glutaminolysis to maintain proliferation and migration in stiff ECM in PAH (34).

### Glu Receptor Signaling in PAH

Glu is an indispensable amino acid that plays a crucial role in signaling as a key excitatory neurotransmitter at the synapses of neurons (46). The functions of Glu are facilitated by the Glu receptors (GluRs), which are categorized into two main groups: ionotropic GluRs (iGluRs) and metabotropic receptors (mGluRs). Many reviews are available on the GluRs in the central nervous system, their functional roles, and how they are implicated in the pathology of neural injury and neuropsychiatric disorders (47–50). However, there is scanty information on its involvement in PAH. GluRs have been characterized based on their sensitivity to specific Glu derivative and their characteristics of the Glu-induced current (Table 2). Structurally, GluR agonists and antagonists are akin to Glu, which allow them to bind the same receptors. A brief overview of GluRs is outlined in Tables 1, 2 because a comprehensive exposition of them has been previously dealt with in detail by others (47–49, 73, 74).

**Table 1:** Receptor subtypes and their localization.

**Receptor subtypes**	**Animal**	**Organ**	**Cell/tissue type**	**References**
GluR 2/3, Ka2, NMDAR1, mGluR5, mGluR 2/3, mGluR1	Rat/monkey	Heart	Atrium/septum, conducting fibers, cardiomyocytes, intercalated disc, ganglia cells, nerve fibers, blood vessels	(51–55)
GluR2/3, Ka2, NMDAR1, mGluR 2/3	Rat/monkey	Ovary and uterus	Corpus luteum, primordial follicles, theca, granulosa cells, oocyte, blood vessels, nerve fibers, ectocervix, myometrium, endometrial glands, epithelium of fallopian tubes	(56)
GluR2/3, Ka2, NMDAR1, mGluR 2/3	Rat	Kidney	Glomeruli, mesangium, podocytes, juxtaglomerular, apparatus, tubules	(55, 56)
GluR 2/3, Ka2, NMDAR1	Rat	Testis	Germinal epithelium, interstitial cells	(53, 56, 57)
GluR2/3, Ka2, NMDAR1	Rat	Gastrointestinal	Enteroendocrine cells, parietal cells of the stomach, pancreatic islets, nerve fibers, ganglia cells, liver	(56, 58–62)
GluR 2/3, Ka 2, NMDAR 1	Rat	Others	Lungs, spleen, megakaryocytes, mast cells, inflammatory cells	(53, 56, 63–66)

**Table 2:** Glutamate receptor protein subunit composition and properties.

**Receptor**	**Receptor properties**
**iGluRs**	
NMDAR	Heterotetramer; ↑Ca^2+^ permeability; long channel open time (67)
AMPAR (α-amino-3-hydroxy-5-methyl-4-isoxazole propionic acid receptor)	Heterotetramer; ↓Ca^2+^permeability low when edited GluR_2_, somewhat moderate; short channel open time (68)
Kainate receptor	Heterotetramer or homotetramer; ↓Ca^2+^ permeability; short channel open time (69–71)
**mGluRs**	
Group 1	Homodimer; signals via phospholipase C; located postsynaptically (72)
Group 2	Homodimer; located mostly presynaptically; signals via adenylyl cyclase (AC); agonists and antagonists mostly distinct from group 3 (72)
Group 3	Homodimer; mostly located presynaptically; signals via AC; agonists and antagonists usually different from group 2 (72)

**Figure 2:**
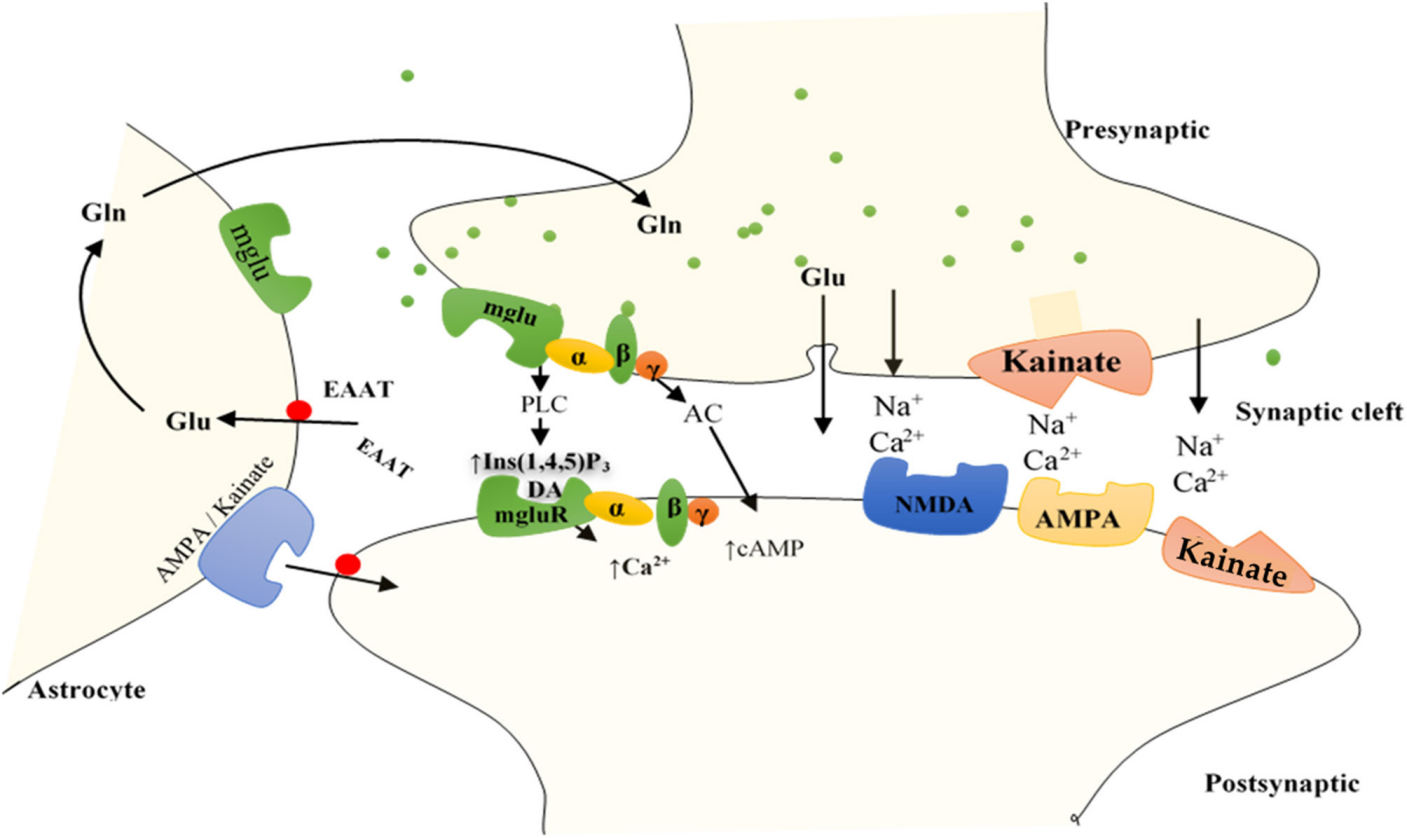
Function of GluRs: Glutamate discharged from presynaptic neurons spread across the cleft of the synapse (by diffusion) to bind to receptors situated on the postsynaptic cell membrane. Group I mGluRs are affixed to PLC, whereas groups II and III receptors trigger the inhibitory cAMP pathway. IGluRs have permeability largely for Na^+^ and Ca^2+^. Discharged glutamate is predominantly removed by specific excitatory amino acid transporters (EAATs) found on neighboring astrocytes. Accumulated glutamate inside the astrocytes is transformed to glutamine and transported to the presynaptic cell, which forms glutamate through mitochondrial glutaminase to be re-released. Astrocytes also express AMPA and kainate IGluRs, including mGluRs, bestowing evidence for bidirectional signaling of glutamate involving neurons and astrocytes. AC, adenylyl cyclase; DAG, diacylglycerol; Gln, glutamine; Glu, glutamate; Ins (1,4,5)P_3_, inositol (1,4,5)-trisphosphate.

The iGluRs contain intrinsic cationic channels linked with ligand-binding sites. The iGluR family is organized into three main subtypes: *N*-methyl-d-aspartate (NMDA), α-amino-3-hydroxy-5-methyl-4-isoxazole propionic acid, and kainate receptors (73). The membrane channels linked with these receptors depict different electrophysiological and pharmacological properties, including ionic channel selectivity to sodium (Na^+^), K^+^, and Ca^2+^ (73, 75) (Figure 2). For instance, the NMDA receptors (NMDARs), found in blood vessels, are permeable to Ca^2+^ ions and have been reported to drive vascular remodeling in PAH (43). Ca^2+^ activates protein kinase C and its downstream effects (AKT/MAPK pathways), which results in increased cell survival and proliferation, a driver of remodeling in PAH. When NMDARs were blocked, it stopped PAH progression and reversed vascular remodeling (43).

Unlike iGluRs, the mGluRs exact their outcomes either on secondary messengers or ion channels via the stimulation of the guanosine triphosphate–binding proteins and regulate the biosynthesis of distinct intracellular secondary messengers, such as triphosphate inositides (IP_3_), cyclic adenosine monophosphate (cAMP), or cyclic guanosine monophosphate (47, 73, 75, 76) (Figure 2). Like iGluRs, the mGluRs are categorized into four groups. Group I, comprising mGluR 1, 5, and 6, induces triphosphoinositides (IP_3_) metabolism and mobilization of intracellular Ca^2+^ ions (77) (Figure 2). mgluR5 couples with G_α*q*/11_ to activate IP_3_ leading to increased release of intracellular Ca^2+^ and its subsequent signaling mentioned above. Group II (mGluR 2 and 3) and group III (mGLuR4, 6–8) are coupled to adenylyl cyclase (AC) (47, 73, 75, 76, 78) (Figure 2). ACs are activated by PGI_2_ to promote vasorelaxation and vasodilation, as well as increase cAMP levels yielding in the activation of protein kinase A (PKA). Activation of PKA resumes various phosphorylating pathways, thereby attenuating the glycolytic shift in PAH. PGI_2_ also attenuates proliferation of vascular SMCs, inhibition of platelet aggregation, and exertion of anti-inflammatory and antithrombotic effects through this same (PKA signaling) pathway (79). As such, both iGluRs and mGluRs may play significant roles in PAH pathogenesis.

### Metabolic Dysregulation and Glutaminolysis as Therapeutic Targets in PAH

Metabolism is an intricate sequence of molecular events that involve the control of several downstream characteristics of PH. However, it is also a closely regulated process, which means therapeutically controlling metabolic pathways in PAH may be difficult and might yield unintended side effects. Conserved metabolic processes might give room for interventions with the capability of extensive targeting in various tissues and cell types in PAH; nonetheless, the effectiveness of aiming at a conserved pathway can be challenging when considering the local vs. systemic administration of a therapeutic intervention. Likewise, the inherent complexity of metabolism portends a challenge when choosing a target effector, which must, in an ideal condition, have a master regulatory function in reprogramming events. The metabolic disparity also serves as a hurdle in treating potential epigenetic, genetic, and PH-variant differences, which confines innovative therapies to particular populations of patients. Regardless of such challenges, therapeutically targeting metabolism holds an enormous promise to treat early PAH, giving room for the deterrence and/or reversion of pathologic cellular phenotypes (80). Glutaminolysis has become a center of attention in most researches investigating metabolic effectors in PAH as it has shown promising therapeutic features in some animal models of PAH. For instance, Piao et al. and other researchers reported an increase in glutamine uptake in human patients and animal models of PAH (36, 38, 43, 81). The inhibition of GLS1 in some clinical trials using cancer drugs (NCT02071862) (82), NMDAR using MK-801(43), and YAP using verteporfin (34) has proven to be beneficial in ameliorating PAH progression.

With recourse to the reliance of cancer cells on glutaminolysis, targeted therapies have been developed against glutamine metabolism from glutamine uptake to enzymes that characterize glutaminolytic reactions. For example, the GLS inhibitors 968, bis-2-(5-phenylacetamido-1,2,4-thiadiazol-2-yl)ethyl sulfide 3 (BPTES) and telaglenastat (CB-839) (in phase 1 clinical trials: NCT02071888, NCT02071862, and NCT02771626) have shown promising tumor-suppressive activities in many preclinical animal models (83). In addition to GLS inhibitors, regimens targeting Glu's conversion into α-ketoglutarate, such as Glu dehydrogenase inhibitors and aminotransferase inhibitors (e.g., amino-oxyacetic acid), have also been assessed in preclinical models of breast cancer and neuroblastoma (83–86). Also, glutamine analogs such as 6-diazo-5-oxo-l-norleucine (l-DON), acivicin, and azaserine were reported to nonspecifically target many glutaminolytic enzymes, thereby attenuating Glu-dependent nucleotide biosynthesis in both preclinical and clinical studies (87). Even though these glutaminase inhibitors and Glu analogs introduced the possibility of targeting Glu addiction in PAH, dose-limiting toxicities associated with their usage, as observed in cancer, must be carefully considered. Despite the dose-limiting toxicities that characterized some glutamine analogs, compounds targeting specific glutaminolytic steps hold enormous therapeutic potential in targeting Glu metabolism in PAH treatment. For instance, the inhibition of the solute carrier family 1 member 5 (SCL1A5), a known glutamine transporter and an indispensable factor for cell proliferation, by l-γ-glutamyl-p-nitroanilide (GPNA) suppressed tumor growth in lung cancer (88). Also, the SCLA5 inhibitors tamoxifen and raloxifene have been reported to restrict glutamine uptake (89). Furthermore, the SLC7A5 inhibitor 2-aminobicyclo(2,2,1)-heptane-2-carboxylic acid (BCH) was reported to inhibit mTOR signaling (90) and hence might be able to attenuate glutaminolysis-induced fibrosis resulting from αKG-mediated mTOR activation and proline hydroxylation in PAH (35).

Finally, the repurposing of presently available therapies that target various metabolic pathways in metabolic disorders such as diabetes might yield beneficial effects in PAH treatment. For example, empagliflozin, a Na^+^-glucose cotransporter 2 (SGLT2) inhibitor, was reported to lower mortality, reduce RV systolic pressure, and attenuated maladaptive pulmonary remodeling in animal models, thereby preventing PAH progression (91). However, it is not known whether empagliflozin and other SGLT2 inhibitors can reverse an already established PAH, as such further studies would be needed to ascertain the spectrum of their action and usage in PAH therapy. Some drugs that are under development or may be repurposed for PAH treatment have been summarized in Table 3. Also, clinical trials targeting RV dysfunction in PAH has been extensively review by Prisco et al. (100).

**Table 3:** Potential repurposable drugs for PAH management.

**Drug**	**Mechanism of action**	**Possible effect on PAH**	**References**
Pioglitazone	Activation of peroxisome proliferator-activated receptor-γ (PPARγ); regulates the expression of various genes tackling insulin resistance, inflammatory changes, and vascular remodeling	Alleviation of cardiac and vascular remodeling; improvement in survival	(92)
Empagliflozin	Sodium–glucose cotransporter 2 (SGLT2) inhibitor; augments excretion of urinary glucose, as well as reduces cardiovascular events and mortality in type 2 diabetes patients	Improve hemodynamics and survival; reduces right ventricular hypertrophy and fibrosis; decreases pulmonary arteriole muscularization and attenuation of maladaptive cardiac remodeling	(91, 93–95)
NTP42	Novel thromboxane prostanoid receptor (TP) antagonist (currently under development for PAH treatment)	Inhibition of excessive vasoconstriction and remodeling of pulmonary artery, *in situ* thrombosis, inflammation and fibrosis	(96)
Spironolactone	Attenuation of impaired vascular reactivity and endothelial dysfunction due to hyperaldosteronemia-induced glucose-6-phosphate dehydrogenase (G6PD) deficiency	Improvement in endothelial dysfunction, vascular reactivity, NO bioactivity and vasodilation; must, however, be used along with drugs with antiremodeling properties for effectiveness	(97, 98)
γ-Folate binding protein (γ-FBP)	SCL5A1 inhibitor; prevention of glutamine transport	Inhibition of glutaminolysis; antiproliferation and antiremodeling; prevention of PAH progression and reversal of an already established diseased condition; suppression of mTOR signaling, thereby attenuating mTOR-mediated fibrosis	(99)
Epigallocatechin gallate (EGCG) (NCT02891538) R162	Glu dehydrogenase inhibitors; prevents the conversion of glutamate to αKG; disrupts anaplerotic utilization of glutamine in the TCA cycle	Suppression/prevention of αKG-induced mTOR activation and proline hydroxylation	(86)

## Conclusion and Future Perspectives

Discussions on the pathophysiology of PAH over the years majorly centered on the nitric oxide–thromboxane–endothelin 1 (NO–TXA_2_-ET_1_) pathways. However, metabolic dysregulation and reprogramming, resulting in a shift from OXPHOS toward glutaminolysis, have been implicated in PAH pathogenesis (34). This shift results in Glu addiction, a condition where cells preferentially depend on glutamine as their substrate source for energy production. Glu addiction results in increased glutaminolysis, which triggers the activation of glutaminolytic enzymes. Activation of glutaminolytic enzymes such as GLS drives arterial remodeling in animal models of PAH, whereas increased glutaminolysis induces fibrosis and ignites aggressive hyperproliferation, arterial stiffening, and ECM matrix migration. Also, glutamatergic communication via GluRs triggers remodeling and obstruction of pulmonary arterioles. Besides, the localization of GluRs such as NMDAR and mgluR5 in the blood vessels and RVs, respectively, alludes to their possible role in PAH pathogenesis. As such, the targeting of Glu metabolism, glutaminolytic enzymes, and GluRs as therapeutic effectors holds enormous potential in PAH therapy. The repurposing of certain drugs used in the treatment of diabetes and other metabolic disorders to target the metabolic aberrations in PAH might also yield beneficial results. However, more research needs to be conducted to elucidate their safety for clinical use to avoid unintended side effects that might result from targeting a metabolic pathway(s).

## Author Contributions

The review idea was conceived by RM. RM, GA, and YG drafted and wrote the manuscript. With the supervision of QW, RM, GA, YG, MN, AA, JA-A, MD, and PW revised and proofread the manuscript. All authors contributed to the article and approved the submitted version.

## Conflict of Interest

The authors declare that the research was conducted in the absence of any commercial or financial relationships that could be construed as a potential conflict of interest.
